# Electronic Health Interventions for Preventing and Treating Negative Psychological Sequelae Resulting From Pediatric Medical Conditions: Systematic Review

**DOI:** 10.2196/12427

**Published:** 2019-11-11

**Authors:** Ashley Brook McGar, Christine Kindler, Meghan Marsac

**Affiliations:** 1 Kentucky Children's Hospital University of Kentucky Lexington, KY United States; 2 College of Medicine University of Kentucky Lexington, KY United States

**Keywords:** telemedicine, children, caregivers, injury, chronic disease, wounds and injuries, depression, anxiety

## Abstract

**Background:**

Pediatric medical conditions have the potential to result in challenging psychological symptoms (eg, anxiety, depression, and posttraumatic stress symptoms [PTSS]) and impaired health-related quality of life in youth. Thus, effective and accessible interventions are needed to prevent and treat psychological sequelae associated with pediatric medical conditions. Electronic health (eHealth) interventions may help to meet this need, with the capacity to reach more children and families than in-person interventions. Many of these interventions are in their infancy, and we do not yet know what key components contribute to successful eHealth interventions.

**Objective:**

The primary objective of this study was to conduct a systematic review to summarize current evidence on the efficacy of eHealth interventions designed to prevent or treat psychological sequelae in youth with medical conditions.

**Methods:**

MEDLINE (PubMed) and PsycINFO databases were searched for studies published between January 1, 1998, and March 1, 2019, using predefined search terms. A total of 2 authors independently reviewed titles and abstracts of search results to determine which studies were eligible for full-text review. Reference lists of studies meeting eligibility criteria were reviewed. If the title of a reference suggested that it might be relevant for this review, the full manuscript was reviewed for inclusion. Inclusion criteria required that eligible studies (1) had conducted empirical research on the efficacy of a Web-based intervention for youth with a medical condition, (2) had included a randomized trial as part of the study method, (3) had assessed the outcomes of psychological sequelae (ie, PTSS, anxiety, depression, internalizing symptoms, or quality of life) in youth (aged 0-18 years), their caregivers, or both, (4) had included assessments at 2 or more time points, and (5) were available in English language.

**Results:**

A total of 1512 studies were reviewed for inclusion based on their title and abstracts; 39 articles qualified for full-text review. Moreover, 22 studies met inclusion criteria for the systematic review. Of the 22 included studies, 13 reported results indicating that eHealth interventions significantly improved at least one component of psychological sequelae in participants. Common characteristics among interventions that showed an effect included content on problem solving, education, communication, and behavior management. Studies most commonly reported on child and caregiver depression, followed by child PTSS and caregiver anxiety.

**Conclusions:**

Previous research is mixed but suggests that eHealth interventions may be helpful in alleviating or preventing problematic psychological sequelae in youth with medical conditions and their caregivers. Additional research is needed to advance understanding of the most powerful intervention components and to determine when and how to best disseminate eHealth interventions, with the goal of extending the current reach of psychological interventions.

## Introduction

### Background

An estimated 6 million children are admitted to hospitals annually in the United States, often under life-threatening circumstances [1]. In addition to the physical impact of pediatric injuries and illnesses, consequences of pediatric medical conditions can often include negative psychological sequelae such as posttraumatic stress symptoms (PTSS), anxiety, depression, and impaired health-related quality of life (HRQoL) for both patients and their caregivers [2-5]. Web-based or electronic health (eHealth) interventions have the potential to mitigate these effects and to extend the reach of in-person interventions [6,7].

Psychological outcomes for acute and chronic conditions are similar [8]. For both acute (eg, burns and traumatic brain injury [TBI]) and chronic medical conditions (eg, atopic dermatitis, chronic pain, cancer, and diabetes), youth and caregivers are at risk for developing significant psychological symptoms and impairment in HRQoL, which have been associated with negative health outcomes. Approximately 30% of children with an injury or illness develop significant PTSS [8]. PTSS post injury is linked to depression, poor health outcomes, and impaired HRQoL [9]. PTSS have been associated with impaired HRQoL and subsequent health problems in youth [10]. In addition, increased anxiety before surgery has been associated with poorer health outcomes and worse postoperative recovery [11]. Similarly, children with chronic conditions, such as cancer, may experience distressing emotional reactions and lower quality of life (QoL), sometimes for years after the completion of treatment [12-14]. Thus, developing effective interventions to address these symptoms and challenges is essential for promoting full recovery (ie, physical and emotional) in youth with medical conditions.

A number of interventions currently exist to promote emotional recovery and adaptation in youth experiencing medical conditions, including education-based interventions, behavioral therapy, cognitive behavioral therapy (CBT), problem-solving therapy (PST), family therapy, multisystemic therapy, and systemic treatment [15-17]. However, limited access to psychologists, high costs of therapy, and difficulty accessing resources because of location (eg, rural areas) are all factors that can play a role in preventing many children from obtaining these treatments [17,18]. eHealth interventions may provide an avenue to distribute evidence-based strategies and treatments to children and families that may not otherwise have access to emotional health resources. Efforts are underway to translate current evidence-based in-person interventions to eHealth platforms.

Multiple reviews have been conducted to examine the use of eHealth interventions to improve health outcomes in children, suggesting promising results for this technology [19-22]. In examining emotional health outcomes, a recent review by Canter et al [23] summarized current evidence for technology-delivered interventions in improving family-centered outcomes (eg, communication, problem solving, and caregiver-child relationship) for children with chronic medical conditions.

### Objectives

Results suggested that although eHealth interventions are generally effective at reducing family conflict, the findings for other family-centered outcomes varied [23]. This review expands upon the findings of the study by Canter et al [23], examining specific individual emotional health outcomes of children and caregivers. Other reviews have examined the impact of eHealth interventions on physical and dietary changes, behavior change, and various health conditions such as asthma but have generally focused on a single medical condition and often only assessed QoL outcomes [19-22]. To our knowledge, no other reviews to date have examined the use of eHealth interventions on psychological outcomes across both acute and chronic pediatric medical conditions.

eHealth interventions can be cost-effective and easily accessible, but their initial development and maintenance can be costly. Without evaluation, it is unknown whether evidence-based, effective interventions are able to maintain their effect when they are translated or adapted for eHealth platforms. Many eHealth interventions are in the early stages of development and have shown promising early results on their efficacy. We conducted this systematic review with the goals of (1) summarizing the state of the field for eHealth interventions that are designed to prevent or treat negative psychological sequelae (ie, anxiety, depression, PTSS, and HRQoL) resulting from pediatric medical conditions and (2) providing recommendations toward future development of eHealth interventions.

## Methods

### Literature Search Strategy

The following databases were searched for all studies conducted between January 1, 1998, and March 1, 2019: MEDLINE (PubMed) and PsycINFO. The search terms used are as follows: “medical trauma,” “medical event,” “medical condition,” “medical procedures,” “illness,” “injury,” “web-based,” “mobile,” “e-Health,” “internet,” “telehealth,” mHealth,” “text,” “application,” “posttraumatic stress symptoms,” “PTSD,” “PTSS,” “posttraumatic stress disorder,” “quality of life,” “anxiety,” “depression”, “psychological sequelae,” ” “intervention,” and “prevention.” Reference lists were reviewed for all studies meeting eligibility criteria. If the title in a reference list suggested that it could be relevant for this review, the full manuscript was reviewed.

### Inclusion and Exclusion Criteria

Eligible studies (1) employed empirical methods examining the efficacy of an eHealth intervention to prevent or treat negative psychological sequelae in youth (aged 0-18 years), their caregivers, or both youth and caregivers as either a primary or secondary outcome, (2) included youth or caregivers of youth with acute or chronic medical conditions, and (3) were available in English language. Studies were excluded if they failed to meet the inclusion criteria listed above. For the purposes of this review, psychological sequelae are defined as PTSS, anxiety, depression, and QoL. Studies that did not include these outcomes as a primary or secondary study outcome for either the child or caregiver were excluded.

### Data Extraction

**Figure 1:**
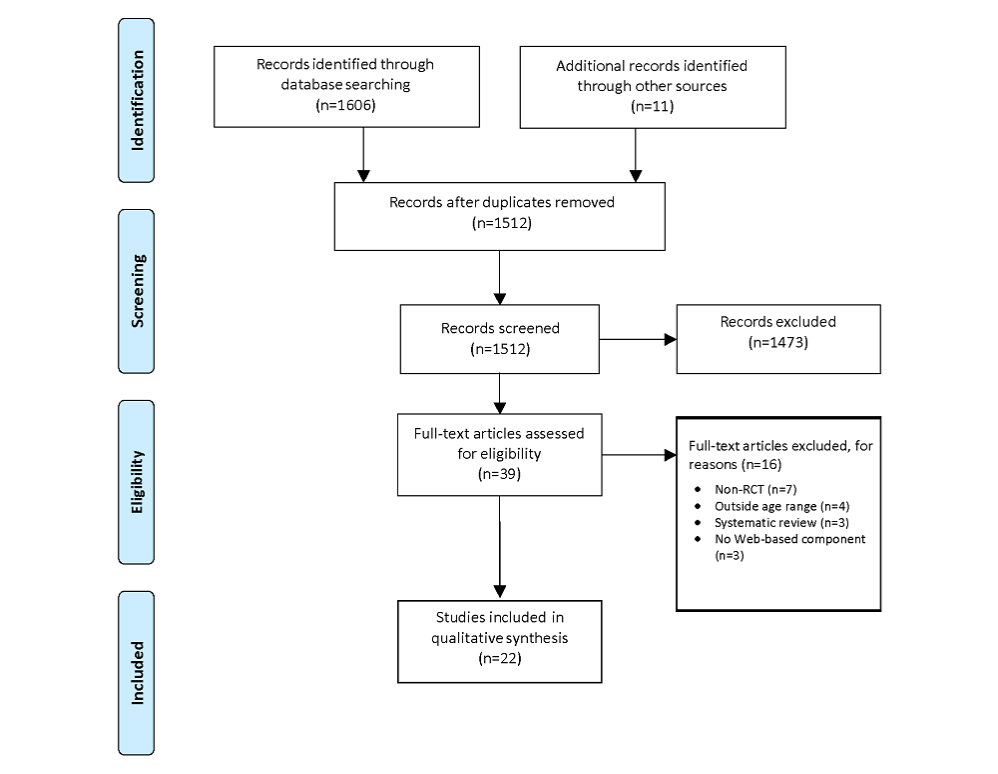
Preferred Reporting Items for Systematic Reviews and Meta-Analyses diagram. RCT: randomized controlled trial.

The initial search of PubMed and PsycINFO yielded 1606 results. A total of 2 authors independently reviewed titles and abstracts of relevant studies focusing on the efficacy of eHealth psychological interventions for preventing or treating psychological sequelae (ie, PTSS, anxiety, depression, and HRQoL) in youth with medical conditions. After removing 105 duplicate studies and excluding 1473 studies based on their title and abstract, the full text of the remaining 39 articles were reviewed for eligibility. A total of 11 additional studies were selected for review from reference sections of the articles in the original search. In total, 22 studies met full inclusion criteria and were included in the review. See Figure 1 for a Preferred Reporting Items for Systematic Reviews and Meta-Analyses diagram depicting the study design of article selection. Each article that met inclusion criteria was reviewed and coded for theoretical framework, intervention delivery method, intervention outcomes, and intervention barriers.

### Checklist for Measuring Study Quality

The Downs and Black checklist was used to assess study quality [24]. The checklist comprises 5 sections (27 items total) measuring reporting, external validity, internal validity, selection bias, and power. Studies were scored and placed into 1 of the 4 categories: poor (>14 points), fair (15-19 points), good (20-25 points), and excellent (26-27 points).

## Results

### Study Quality

The majority of studies scored within the *fair* (50%, 11/22) and *good* (41%, 9/22) categories [25-45]. Only 2 studies [46,47] were rated as *poor* (9%, 2/22). No studies were rated as *excellent*. For the 2 studies rated as *poor*, a score of 0 was given for many items in the internal validity subscale (eg, either did not address or failed to blind participants and researchers, did not address participants lost to follow-up, and did not adjust for confounding in the analyses). Approximately 64% (14/22) of the included studies described characteristics of the participants that were lost to follow-up; 68% (15/22) of the studies accounted for this loss in analyzing study outcomes.

### Study Characteristics

Of the 22 studies included in this review, 8 assessed for depression [27,33-36,40,41,43], 5 for anxiety [27,33,40,41,44], 4 for PTSS [27,31,39,45], and 10 for HRQoL [25,26,29,30,32, 37,38,44,46,47]. A total of 5 studies [27,33,40,44,45] examined more than 1 outcome. All included studies were randomized controlled trials (RCTs) per inclusion criteria. Some of the studies (n=12) [25-27,30,31,34,37-39,44-47] examined interventions that were solely delivered via Web, whereas 10 studies [29,32,33,35,36,38,40-43] used a combination of eHealth program components and sessions with a therapist or coach. See Multimedia Appendix 1 for additional study details.

### Study Participants

Sample sizes for the studies ranged from 37 to 164. Child participants were aged 2 to 18 years. A total of 7 interventions were designed for children alone [25,26,31,32,34,37,46], 3 for caregivers alone [36,39,45], and 11 for children and caregivers to use together [27,29,30,33,35,38,40-42,44,47]. Moreover, 10 studies focused on chronic conditions such as asthma [30,37,47], chronic headache [33], juvenile idiopathic arthritis (JIA) [25,38], inflammatory bowel disease [26], complex congenital heart disease [32], chronic respiratory condition [34], and type-1 diabetes [46]. In addition, 6 studies focused on pediatric patients suffering from psychological sequelae following a TBI [35,36,40-43]. A total of 3 studies focused on the aftermath of injuries (not focused on TBI) [27,39,45], and 1 study included children with potentially traumatic medical events (eg, injury, new diagnosis, and exacerbation of chronic condition) [31]. Furthermore, 2 studies had additional relevant criteria: one study required that participants perceived their injury as potentially traumatic [31], and another study enrolled participants who medical staff perceived as socially isolated or disadvantaged [34].

#### Model and Theoretical Framework of Interventions

A total of 8 eHealth interventions [25,27,29,31,33,38,39,45] used CBT as the basis for informing intervention content. Moreover, 7 [34,35,40-43,46] eHealth interventions used PST as the basis for informing intervention content. In addition, 5 studies [26,30,32,37,47] used education focused on disease management without an additional framework. A total of 2 interventions [27,44] primarily used psychoeducation to inform intervention content. Only 1 study included parent-child interaction therapy (PCIT) [36].

#### Problem Solving

A total of 7 studies [34,35,40-43,46] used PST as the primary framework for interventions. PST focuses on identifying problems, creating new strategies to address problems, and learning ways to implement those strategies [40]. Of the 7 studies, 5 were conducted in children with TBI [35,40-43]. The other 2 included children with type-1 diabetes and chronic respiratory conditions [34,46]. Of the 7 studies, 5 reported achieving at least one targeted outcome and included the following: (1) initial in-person visit with a therapist to introduce the intervention, (2) self-guided Web sessions accompanied by videoconferences with a therapist upon session completion, and (3) a videoconference with a therapist at the end of the intervention to practice learned skills and discuss needs for supplemental sessions [35,40-43]. For example, Petranovich et al developed a Web-based Counselor-Assisted Problem-Solving intervention aimed at identifying problem areas and learning new strategies to address TBI-related challenges [35].

#### Cognitive Behavioral

A total of 7 studies [25,29,31,33,39] used either CBT alone or psychoeducation based in a CBT framework [38,45] to inform intervention content. The primary concept of CBT is to use thoughts and behaviors to modify challenging emotions [48]. Likewise, primary goals of CBT eHealth interventions were to promote adaptive cognitive appraisals [31], normalize reactions to trauma [27], and apply new behavioral strategies [38,45]. In addition, 3 interventions [31,39,45] were designed for children with acute medical events or injuries, 2 [25,38] targeted children with JIA, 1 [33] was designed for chronic headache, and 1 [29] for head or abdominal pain. A total of 6 interventions [27,29,33,38,39,45] provided information through websites and 1 [31] through an interactive game-based format. Of the 7 studies, 3 [27,31,33] reported achieving at least one intended outcome. For example, Cox et al [27] targeted trauma reactions in children with unintentional injury through a Web-based psychoeducation intervention. The intervention website for children contained information on relaxation, coping tips, problem solving, and other cognitive behavioral strategies. Caregivers were provided with an informational booklet containing tips on how to help their child recover after experiencing trauma [27]. Only 2 [29,38] of the 7 CBT interventions involved weekly telephone or email meetings with a trained coach to review intervention materials and answer questions. Although these interventions were based primarily on a CBT framework, interventions were multifaceted and included problem-solving elements and educational elements.

#### Parent-Child Interaction Therapy

A single intervention, developed by Raj et al, was based in a PCIT framework [36]. PCIT aims to improve the relationship between caregivers and children by teaching caregivers how to best respond to their child’s behavior, listen effectively, and encourage their child’s efforts to improve [49]. Raj et al [36] designed an intervention that combined traditional PCIT with additional stress and anger management to support caregivers of children with TBI. The intervention comprised 10 eHealth sessions delivered in 2 parts each: self-guided (part 1) and videoconference with a therapist (part 2). Caregivers received education on topics such as positive thinking, stress and behavior management, and disciplining their child after TBI [36]. This intervention also combined elements of other frameworks (ie, cognitive behavioral, education, and behavior management) with PCIT to provide caregivers with new skills for dealing with challenges following pediatric TBI. This intervention did not achieve significant outcomes.

#### Psychoeducation

Psychoeducation was a key component for 2 interventions [27,44]. These interventions focused on educating, normalizing, and relieving anxiety or trauma reactions. Both of these interventions achieved at least one targeted outcome. For example, the intervention by Fortier et al [44] provided education, skills training, and interactive games via a website to prepare children and their caregivers for what to expect before, during, and after surgery, focusing specifically on managing anxiety and pain.

#### Education Only

Several studies [26,30,32,37,47] used health education–only theoretical frameworks for their interventions. Out of these 5 interventions, 3 [30,37,47] used the following self-management components: disease education, self-monitoring, creating an action plan, and regular medical review. Of these 5 interventions, 3 [30,37,47] achieved at least one targeted outcome. For example, Klausen et al [32] used health education to increase perceived competence in patients with complex congenital heart disease and provide behavioral change techniques. This included information on the benefits of physical exercise, how to set goals and create an action plan, identifying barriers and problem solving, environmental structuring, social comparison, time management, and providing future rewards [32].

#### Electronic Health Intervention Delivery Methods

All interventions were delivered in a Web-based format. For many studies (n=10) [26,31,32,35,36,40-43,45], therapists introduced the intervention to participants during an initial in-person meeting. The majority of interventions with a therapist or coach component [29,35,36,40,41,43,45,47] included ongoing contact (eg, phone, email, or videoconferencing) to review intervention content and discuss supplemental materials. Some eHealth interventions [27,33,34,37,44,46] were used with full independence by study participants or with extra contact being limited to emails encouraging intervention use [45]. In 1 intervention [39], therapists provided written feedback on weekly homework assignments. Moreover, 1 study [25] used a combination of internet and individual instruction but gave no specific details on how individual instruction was provided. Most interventions were initiated after the child was discharged or had completed initial medical treatment. Only 1 intervention [27]—an eHealth intervention intended to provide information to parents after an unintentional pediatric injury to prevent or address early PTSS—was initiated while the child was undergoing medical treatment and then continued post discharge from hospital care.

### Description of Outcomes

Research varied in whether outcomes were assessed for children only (n=7 studies) [25,26,31,32,34,37,46], caregivers only (n=3 studies) [36,39,45], or both children and caregivers (n=12 studies) [27,29,30,33,35,38,40-44,47]. Of the included interventions, 13 [27,30,31,33,34,37,39-44,47] identified significant intervention effects on at least one targeted psychological outcome.

#### Child Outcomes

Of the 7 studies that assessed child outcomes, 5 [27,33,34,41,42] focused on depression. Although Law et al [33] saw a brief improvement in child depression in children with chronic headaches at 3-month follow-up in both groups, no significant differences were seen between groups post intervention. Only when moderating factors were included, did some of these interventions show an effect [41,42]: after controlling for family socioeconomic status (SES) as a moderator in analyses, Wade et al [40] and Wade et al [29] found a decrease in depressive symptoms in the intervention group compared with the control group.

Interventions targeting child anxiety [27,33,41,42,44] were more successful, with 4 [27,41,42,44] of the 5 studies showing improvement in anxiety post intervention. For example, Cox et al [27] designed an intervention for youth suffering from anxiety following an unintentional injury and offered coping strategies (eg, relaxation, coping statements, and problem solving) and a booklet containing information about the caregiver’s role in the child’s recovery process. Results indicated that children in the intervention group reported significantly less anxiety at a 5-month follow-up assessment compared with the control group [27].

Of the 3 studies that evaluated the impact of eHealth interventions on child PTSS, 2 [31,45] found a statistically significant improvement of symptoms post intervention. These programs primarily focused on normalizing PTSS and offered practical strategies (eg, identifying feelings, relaxation, coping statements, and working through avoidance of trauma reminders) through both text- and game-based activities. The intervention that failed to detect a statistically significant reduction in PTSS had a small sample size, which may have limited their power to detect an effect [27].

A large majority (n=7) of interventions aimed at improving HRQoL in children with chronic illness (eg, chronic respiratory conditions, chronic pain, JIA, inflammatory bowel disease, heart disease, and type-1 diabetes) focused on education about the disease and promoted self-management strategies [26,30,32,37,47] Only 2 interventions targeting child HRQoL found significant differences post intervention, both focused on persistent asthma [30,37]. Although one intervention [37] used internet-based self-management and another [30] used internet-based multimedia asthma education, both studies utilized an interactive asthma monitoring system.

#### Caregiver Outcomes

A total of 4 studies [35,36,40,43] assessed caregiver depression, 2 [40,44] assessed caregiver anxiety, 3 [27,39,45] assessed caregiver trauma symptoms, and 1 [44] assessed caregiver QoL. Although all of the interventions that targeted caregiver depression were designed for children who had experienced a TBI, only 2 [40,43] out of 4 found significant effects.

Both interventions [40,44] aimed at reducing caregiver anxiety had significant effects. Wade et al found a significant decrease in anxiety in caregivers of children with a TBI after completing Web modules and videoconference sessions with a therapist, compared with caregivers who were only provided with internet resources regarding TBIs [40]. Fortier et al [44] provided a Web-based, tailored behavioral preparation program to children who were about to receive surgery and their caregivers and found that caregivers in the intervention group reported significantly less anxiety than those in the control group.

None of the 3 studies [27,39,45] that specifically examined the role of eHealth interventions in preventing or addressing parent posttrauma reactions after a pediatric medical event identified an intervention effect. These interventions were focused on children recovering from unintentional injuries and sought to address posttrauma reactions by providing parents with information about common psychological consequences following a pediatric injury.

#### Barriers Impacting the Efficacy and Usage of Electronic Health Interventions

Of the 22 studies included in the review, 5 [35,36,40,42,43] assessed potential intervention barriers. Of these studies, only 2 [36,40] identified significant moderating effects for potential intervention barriers (eg, SES and level of education). Specifically, Wade and Wolfe [40] suggested that caregivers with a higher income reported greater improvements from intervention use than those with a lower income. Raj et al [36] examined caregiver income and found a significant decrease in global psychological distress from baseline to follow-up for lower-income families. Furthermore, they found that only half of the lower-income families owned a computer and had internet access [36].

## Discussion

### Principal Findings

This review identified 22 studies of eHealth interventions designed to prevent or alleviate negative psychological sequelae (ie, anxiety, depression, PTSS, and HRQoL) in youth with a medical condition and their caregivers. This is this first review to examine multiple psychological sequelae components across acute and chronic medical conditions. By better understanding existing interventions across populations, we can improve our ability to design evidence-based, tailored interventions to improve targeted outcomes.

Overall results from this review indicate that research on eHealth interventions is in early stages and that results are mixed but promising. Results from this systematic review highlight some evidence to suggest that using eHealth interventions may help improve child and caregiver psychological outcomes (including anxiety and PTSS) and functional outcomes (ie, HRQoL) [27,30,31,33,37,40-44,47] but that more research is necessary to examine essential intervention targets, variable needs of different medical populations, and barriers to intervention implementation. In addition, there is room for improvement on designing research studies to thoroughly evaluate the selected outcomes and to explicitly report study methods, as only 41% of studies included were coded within the *good* range of study quality. As many of the studies included in this review were early stage research of new interventions, limitations to study quality are reasonable, as it makes sense to first examine intervention potential before investing in larger, complex research. However, to improve the scientific rigor of the methodology of this research as it continues to grow, future researchers may want to consider carefully examining and reporting on intervention compliance, participant dropout, and the effect of cofounders on outcomes. In addition, future studies may want to aim for larger sample sizes to improve power, the inclusion of larger proportions of the targeted population in the study, and the implementation of double blinding.

As there were very few studies that examined the effectiveness of eHealth studies on child and caregiver emotional health and QoL and as there was substantial variability in intervention components and modality, populations in which interventions are delivered, intervention modality, and outcomes assessed, the following summaries of key findings should be interpreted as preliminary. Results from this systematic review suggest that the intervention theory that guided intervention content might have influenced the outcomes: of the studies included in this systematic review, those that primarily utilized CBT (80%) had the highest proportion of achieving at least one intended outcome [31,33,39]. This was followed by problem solving (71%) [34,40-43], education alone (60%) [30,37,47], psychoeducation (50%) [27-44], and PCIT (0%) [36]. These findings are consistent with current research supporting CBT [50-52].

In examining more nuanced study results, findings suggest that the type of intervention needed may be dependent upon the situation and goal of the intervention (ie, prevention vs treatment of symptoms). For example, information provision or educational interventions appear to be useful for decreasing anxiety and improving QoL, such as preoperative education, or when interventions are delivered within the hours after experiencing trauma (eg, injury) to prepare families for the emotional and physical challenges that lie ahead [27,44]. However, education-based interventions may not be helpful in achieving targeted outcomes for chronic illnesses (eg, JIA, inflammatory bowel disease, and congenital heart disease) [26,32,38] or if the education is provided days after a trauma [39,45]. Thus, intervention modality may need to be selected based on the challenges presented and targeted outcomes.

Results also suggest that intervention modality was fairly comparable regardless of whether the intervention was fully Web-based or not (64% [14/22] indicated significant positive outcomes), compared with Web-based plus in-person contact (44%). This is promising in that fully Web-based interventions may be less costly and have wider reach.

### Potential Intervention Barriers

There are multiple factors to consider that have the potential to interfere with Web-based intervention engagement and effectiveness. Our results indicate that demographic factors such as SES, level of caregiver education, and social advantage may impact intervention efficacy [36,40]. How SES affects technology-based intervention uptake remains mixed. Although low SES is a potential barrier to intervention efficacy, some studies reported that families of lower SES might equally benefit from eHealth interventions compared with families of higher SES, when provided with computers and internet access [35,36,40,42,43]. More research is needed to better understand how intervention can be tailored to be the most efficacious across family SES.

Another potential barrier to consider is knowledge of technology. Although not included in this review because primary results are presented in another publication [40], a study by Carey et al [53] assessed past experience with technology in participants completing the intervention and found that participants who used technology less frequently showed less improvement in anxiety and depression symptoms, compared with those who used technology more often. The wide range of technology experience in target pediatric populations and their parents can been explained by the *digital divide*, defined as the gap in the frequency of information technology use and what it is used for, which is thought to be moderated by demographic factors (eg, income, education level, gender, and race) [53]. Jackson et al [54] surveyed 515 children and found that children of parents with a full-time job used cell phones more frequently than children of parents with other employment situations. In addition, children of parents with higher education reported more use of computers and internet [54]. Female participants used cell phones more often than males, whereas males most intensively used video games [54]. Furthermore, African American males were the least frequent users of computers and internet, whereas African American females were the heaviest users of internet out of all groups [54]. For these reasons, care should be taken when designing interventions to provide the target sample with a feasible intervention platform (eg, adding more or less directions, using pictures, and content delivery method). Future studies should take into account demographical differences when formulating intervention content and deciding which delivery method to use (ie, mobile phone app, internet, or video game).

Barriers to intervention compliance should also be considered. Although not included in the final review, Worthen-Chaudhari et al [55] reported on barriers to study compliance and found that participants who dropped out of the study had discontinued medical care, faced problems with internet access, busy schedules, and experienced co-occurring illness during the study. Although these factors were not assessed after completion of the intervention, it is important to consider these as potential barriers to intervention usage that could affect study results.

Future research should directly examine potential intervention barriers such as SES, education level, location, knowledge of technology, and severity of medical condition in youth with various medical conditions. In addition, although existing literature does not indicate how the timing of the intervention affects its efficacy, this may be an important factor to explore in future research. Studies should also aim to clearly identify their treatment outcomes and create interventions designed specifically to improve those outcomes. Finally, additional studies should examine the impact of improvements in parent outcomes on child outcomes.

### Limitations

There are several notable limitations to this study that should be considered in interpreting and generalizing study results. Many studies reviewed had small sample sizes, with limited power as a result. In addition, research studies included were inconsistent in the measures used and the outcomes assessed, and many studies were not RCTs. Moreover, this study was unable to review research that was not published in English. Finally, the majority of the studies published identified an effect for at least one outcome; it is unknown whether these studies represent the majority of studies conducted in this area or whether there are a number of unpublished nonsignificant findings to take into account.

### Conclusions

eHealth interventions have the capacity to broaden our reach to improve emotional health in families with children undergoing medical treatment. Although results are mixed, the results of this study suggest that eHealth interventions may be useful for improving psychological sequelae in pediatric populations with medical conditions such as TBI and other potentially traumatic injuries. More research is needed to identify the most important intervention components and how to ensure that these components are maintained in the translation to eHealth modalities.

## Appendix

Multimedia Appendix 1 Summary of study characteristics and findings (only outcomes included in this review are reported in this table; studies might have assessed additional outcomes; and effect sizes are reported when available).

## Abbreviations

CBT: cognitive behavioral therapy
HRQoL: health-related quality of life
JIA: juvenile idiopathic arthritis
PCIT: parent-child interaction therapy
PST: problem-solving therapy
PTSS: posttraumatic stress symptoms
QoL: quality of life
RCT: randomized controlled trial
SES: socioeconomic status
TBI: traumatic brain injury
