# Supplementary material for: Electronic Health Interventions for Preventing and Treating Negative Psychological Sequelae Resulting From Pediatric Medical Conditions: Systematic Review
Source: JMIR Pediatr Parent. 2019 Nov 11;2(2):e12427. doi: 10.2196/12427 (PMC6878107; doi:10.2196/12427)
Supplement: Multimedia Appendix 1 [file pediatrics_v2i2e12427_app1.pdf]

Table 1. Summary of study characteristics and findings (only outcomes included in this review are reported in this table; studies might have assessed additional outcomes; and effect sizes are reported when available).

Reference	Child medical condition	Child age (years)	Intervention group (n; comparison group (n)	Model/theoretical framework	Intervention content and delivery modality	Outcomes assessed	Downs and Black score	Results
**Family interventions**								
Chan et al (2007) [47]	Persistent asthma	6-17	Virtual group (60); standardized office-based care (60)	Education	Daily completion of an online asthma symptom diary; submission of a video of peak flow measure-ments twice per eek for 6 weeks; phone or mail contact with case manager 2 times per week for 6 weeks, then once per week after	Caregiver and child HRQoL[a]	14	HRQoL improved significantly for caregivers in both groups, from 5.7 (SD 1.1) to 6.4 (SD 1.0) for caregivers in the virtual group and from 5.5 (SD 1.1) to 6.2 (SD 0.8) for caregivers of children in the office-based group (*P*<.05; 2-way analysis of variance). There was no change in HRQoL for children in either group

Cox et al (2010) [27]	Unintentional injury within last 72 hours	7-16	Information provision intervention (29); assessment only (27)	Psychoeducation based on cognitive and resiliency theory; secondary trauma prevention	Booklet for parents; website for children; encouraged to use as needed	Child anxiety, PTSS[b], and depression; caregiver PTSS	22	Children in the intervention condition reported significantly less anxiety at a 5-month follow-up compared with the control condition (Cohen *d*=0.34). No significant differences in depression or PTSS between groups for children or caregivers were detected
Fortier et al (2015) [44]	Outpatient elective surgery	2-7	WebTIPS (38); standard of care (44)	Psychoeducation	Web-based, tailored behavioral preparation program	Child and caregiver preoperative anxiety; HRQoL	21	Children in the WebTIPS group (mean 36.2, SD 14.1) were less anxious than children in the standard of care group (mean 46.0, SD 19.0) at entrance to

								the operating room (*P*=.02; Cohen *d*=0.59) and introduction of the anesthesia mask (mean 43.5, SD 21.7 vs mean 57.0, SD 21.2, respectively; *P*=.01; Cohen *d*=0.63). Parents in the WebTIPS group (mean 32.1, SD 7.4) experienced less anxiety compared with parents in the control group (mean 36.8, SD 7.1) in the preoperative holding area (*P*=.004; Cohen *d*=0.65)

Hicks et al (2006) [29]	Head or abdominal pain	9-16	Internet-based treatment (25); standard medical care waitlist (22)	CBT[c]	Web-based manual for children and parents; weekly therapist contact (phone or email)	Child HRQoL	20	No significant differences in HRQoL
Jan et al (2007) [30]	Persistent asthma following the Global Initiative for Asthma clinical practice guidelines	6-12	Blue Angel for Asthma Kids (88); traditional asthma care (76)	Education	Internet-based multimedia asthma education with interactive asthma monitoring system, 12 weeks	Child HRQoL	16	Both groups reported improvement in QoL[d], but only caregivers in the intervention group reported significant increase in child QoL, from 6.5 (SD 0.5) at baseline to 4.3 (SD 1.2) after the intervention on a 7-point scale (*P*<.05)
Law et al (2015) [33]	Chronic headache	11-17	Internet CBT and specialized headache treatment (44); specialized headache treatment alone (39)	CBT	Internet-delivered family-based CBT intervention	Adolescent anxiety and depressive symptoms	18	Both groups demonstrated a significant decrease in depressive symptoms from baseline to 3-month follow-up (main

								effect for time $F_{2,98}$=5.91; P=.004). There was no statistically significant difference between the internet CBT group and specialized headache treatment group post intervention. There was no statistically significant change in anxiety from baseline to postintervention or follow-up within either group ($F_{2,105}$=1.40; P=.25) or between groups (group×time interaction $F_{2,103}$=0.71; P=.50)

Petranovich et al (2015) [35]	Moderate-to-severe TBI[e] in the past 1-7 months	12-17	CAPS[f] (65); received access to online resources for TBI (67)	PST[g]	1 in-person session, 7 Web-based modules, almost 5 videoconferencing sessions; up to 4 additional sessions provided based on need	Caregiver depression	25	No significant differences in depressive symptoms were identified
Wade et al (2006) [40]	Moderate-to-severe TBI in the past 1-24 months	5-16	Online family problem solving (20); internet resources for TBI (20)	PST	1 in-person family session in home (initial visit), 14 Web-based sessions, videoconference with therapist every 1-2 weeks	Caregiver depression, anxiety	17	Caregivers in the intervention group reported significantly lower depression and anxiety compared with those in the control group at follow-up. Partial eta^2=0.08 to 0.16
Wade et al (2015) [41]	Mild-to-severe TBI in the past 1-6 months	12-17	CAPS (65); received access to online resources for TBI (67)	PST	1 in-person session, 7 Web-based modules, almost 5 videoconferencing sessions; up to 4 additional sessions provided	Child internalizing symptoms (including anxiety and depression)	17	For high-school age participants, significantly lower internalizing symptoms reported at 18-month follow-up for those in

					based on need			the CAPS intervention compared with the those in the control group
Wade et al (2011) [42]	Moderate-to-severe TBI in the past 3-19 months	11-18	TOPS[h] (16); received access to online resources for TBI (19)	PST	1 in-person session, 10-14 Web-based sessions, videoconference with therapist	Child internalizing symptoms	19	For teens with severe TBI, greater improvements in internalizing symptoms were reported for TOPS group compared with control group at an 8-month follow-up (partial eta^2=0.34).
Wade et al (2012) [43]	Moderate-to-severe TBI in the past 3-19 months	11-18	TOPS (20); received access to online resources for TBI (21)	PST	1 in-person session, 9-13 2-part sessions (part 1: self-guided Web-based session; part 2: videoconference with therapist)	Caregiver depression	20	For parents with lower SES[i]: Parents in TOPS group reported significant reductions in depression from baseline to follow-up.

Stinson et al (2010) [38]	JIA[j]	12-18	Web-based intervention (22); control: weekly phone calls with RA[k] to discuss how participant manages their JIA; RAs did not give self-manage information (24)	Psychoeducation based in a CBT framework	12-week period; 12 modules for adolescents (asked to log on once per week; 20-30 min to complete), 2 modules for caregivers; weekly phone calls with coach (RA)	Child HRQoL	15	No significant differences were found between groups for HRQoL
**Caregiver-only interventions**								
Marsac et al (2013) [45]	Unintentional injury sustained within last 60 days	6-17	After the injury (50); received care as usual (50)	Psychoeducation based in a CBT framework	Web-based, self-guided intervention for caregivers; offers videos, quiz, tips	Child and caregiver PTSS	20	No significant group differences were detected for child or caregiver PTSS.
Raj et al (2015) [36]	TBI admitted to hospital for treatment	3-9	I-InTERACT (20); received access to online resources for TBI (17)	Parent-child interaction therapy	1 in-person session; 9 2-part Web-based sessions (part 1: self-guided Web-based session; part 2: videoconference with therapist); 4 supplementa	Caregiver depression	19	No significant differences were found between groups for depression. Barriers reported to utilizing eHealth[l] interventions: rural location, no reliable

					l sessions offered			access to high-speed internet, and low level of education (associated with low SES)
Sveen et al (2017 [39]	Burns	<18	Intervention group (52); waitlist control group (52)	CBT	Internet-based information and support program; 6 modules, 1 module per week, homework assignment once a week	Parent PTSS	16	Parent PTSS briefly improved, but these changes did not maintain over time. The intervention group had lower scores compared with the control group at time 2 (beta=−11.5; SE=3.88; *P*=.003) and time 3 (beta=−7.89; SE=3.38; *P*=.02), but no significant differences were found at baseline or time 4
**Child-only (pediatric patients) interventions**								

Armbrust et al (2017) [25]	JIA	8-13	Rheumates@Work (28); control group (21)	CBT	Internet-based and individual instruction, 14-week cognitive behavioral program, 4 group sessions for improved physical activity	Child HRQoL	18	There were no changes in HRQoL in either group
Carlsen et al (2017) [26]	Inflammatory Bowel Disease	10-17	eHealth: young.constant-care.com (27); control group (26)	Education	2-year intervention; used Web app monthly, seen at 1 preplanned outpatient visit	Child HRQoL	23	No differences were found in QoL
Kassam-Adams et al (2016) [31]	Acute medical event within last 2 weeks; child perceived event as potentially traumatic based on screener	8-12	Coping Coach (36); intervention given upon completion of 12-week follow-up (36)	CBT	Game-based; 4 characters with 3 levels each (level 1: identify feelings; level 2: connecting feelings, thoughts, and behaviors; and level 3: working through avoidance to trauma reminders)	Child PTSS	23	Group differences were identified between the intervention and control groups for reductions in child PTSS from baseline to 6- (d=−0.68) and 12- (d=−0.55)

								week follow-ups. PTSS in waitlist participants remained stable from baseline to 6 weeks, with a small increase in PTSS from baseline to 12 weeks. A small effect size was found for the waitlist group (postintervention) from baseline to 18 weeks (*d*=−0.27)
Klausen et al (2016) [32]	Complex congenital heart disease	13-16	Intervention group (PReVail; 81); control group (77)	Education	eHealth intervention, 45 min of group-based health education, 15 min of individual counseling involving patients' parents	Generic child HRQoL and disease-specific child HRQoL	19	No significant changes were found in HRQoL
Newcombe et al (2012) [34]	Chronic respiratory condition	10-17	BEO_m (19); waitlist control (20)	PST	Internet-based problem-solving program with weekly	Child depression	20	No significant group differences in depression.

					structured modules and an online community			However, after performing analyses accounting for group differences on participants' change scores (ie, difference in scores between T1 and T2), differences across time for each of the BEO and waitlist groups separately, and a priori levels of clinically significant (as opposed to statistically significant) change, those in the BEO group reported significantly lower depression (*P*=.04) compared with baseline scores

Newton and Ashley (2013) [46]	Type-1 diabetes	13-18	Web-based intervention (25); standard medical care (25)	PST	Interactive, 7-week Web-based intervention; discussion forums, chat rooms, and blogs	Diabetes-related child QoL	13	There were no significant differences between the intervention and control groups for QoL
Rikkers-Mutsaerts et al (2012) [37]	Persistent and not well-controlled asthma	12-18	IBSM[n] (46); usual care (44); waitlist group (34)	Education	IBSM; weekly asthma control monitoring with treatment advice	Asthma-related child QoL	16	HRQoL improved for the intervention group at 3 months by 0.40 points on the Pediatric Asthma Quality of Life Questionnaire (95% CI 0.17-0.62; *P*<.01), compared with 0.0 points for the those who received usual care (*P*=.02 for the difference). At 12 months, the difference between the groups was −0.05

								(95% CI −0.05 to 0.41; *P*=.85)

[a]HRQoL: health-related quality of life.

[b]PTSS: post-traumatic stress symptoms.
[c]CBT: cognitive behavioral therapy.
[d]QoL: quality of life.
[e]TBI: traumatic brain injury.
[f]CAPS: Counselor-Assisted Problem Solving.
[g]PST: post-traumatic stress.
[h]TOPS: Teen Online Problem Solving.
[i]SES: socioeconomic status.
[j]JIA: juvenile idiopathic arthritis.
[k]RA: research assistant.
[l]eHealth: electronic health.
[m]BEO: Breathe Easier Online.
[n]IBSM: internet-based self-management.

References

25. Armbrust W, Bos GJFJ, Wulffraat NM, van Brussel M, Cappon J, Dijkstra PU, et al. Internet program for physical activity and exercise capacity in children with juvenile idiopathic arthritis: a multicenter randomized controlled trial. Arthritis Care Res 2017;69(7):1040-1049.

26. Carlsen K, Jakobsen C, Houen G, Kallemose T, Paerregaard A, Riis LB, et al. Self-managed eHealth disease monitoring in children and adolescents with inflammatory bowel disease: a randomized controlled trial. Inflamm Bowel Dis 2017;23(3):357-365.

27. Cox C, Kenardy J. A randomised controlled trial of a web-based early intervention for children and their parents following accidental injury. J Pediatr Psychol 2010;35:581-592.

29. Hicks CL, von Baeyer CL, McGrath PJ. Online psychological treatment for pediatric recurrent pain: a randomized evaluation. J Pediatr Psychol 2006;31(7):724-736.

30. Jan RL, Wang JY, Huang MC, Tseng SM, Su HJ, Liu LF. An internet-based interactive telemonitoring system for improving childhood asthma outcomes in Taiwan. Telemed J E Health 2007;13(3):257-268.

31. Kassam-Adams N, Marsac ML, Kohser K, Kenardy J, March S, Winston FK. Pilot randomized controlled trial of a novel web-based intervention to prevent posttraumatic stress in children following medical events. J Pediatr Psychol 2016;41 138-148.

32. Klausen SH, Andersen L, Sondergaard L, Jakobsen JC, Zoffmann V, Dideriksen K, et al. Effects of eHealth physical activity encouragement in adolescents with complex congenital heart disease: The PReVaiL randomized clinical trial. Int J Cardiol 2016;221:1100-6.

33. Law EF, Beals-Erickson SE, Noel M, Claar R, Palermo TM. Pilot randomized controlled trial of internet-delivered cognitive-behavioral treatment for pediatric headache. Headache 2015;55(10):1410-1425.

34. Newcombe PA, Dunn TL, Casey LM, Sheffield JK, Petsky H, Anderson-James S, et al. Breathe Easier Online: evaluation of a randomized controlled pilot trial of an Internet-based intervention to improve well-being in children and adolescents with a chronic respiratory condition. J Med Internet Res 2012;14(1):e23-e23.

35. Petranovich CL, Wade SL, Taylor HG, Cassedy A, Stancin T, Kirkwood MW, et al. Long-term caregiver mental health outcomes following a predominately online intervention for adolescents with complicated mild to severe traumatic brain injury. J Pediatr Psychol 2015;40(7):680-688.

36. Raj SP, Antonini TN, Oberjohn KS, Cassedy A, Makoroff KL, Wade SL. Web-based parenting skills program for pediatric traumatic brain injury reduces psychological distress among lower-income parents. J Head Trauma Rehabil 2015;30(5):347-356.

37. Rikkers-Mutsaerts ER, Winters AE, Bakker MJ, van Stel HF, van der Meer V, de Jongste JC, et al. Internet-based self-management compared with usual care in adolescents with asthma: a randomized controlled trial. Pediatr Pulmonol 2012;47(12):1170-1179.

38. Stinson J, McGrath P, Hodnett E, Feldman B, Duffy C, Huber A, et al. Usability testing of an online self-management program for adolescents with juvenile idiopathic arthritis. J Med Internet Res 2010;12(3):e30.

39. Sveen J, Andersson G, Buhrman B, Sjoberg F, Willebrand M. Internet-based information and support program for parents of children with burns: a randomized controlled trial. Burns 2017;43(3):583-591.

40. Wade SL, Carey J, Wolfe CR. An online family intervention to reduce parental distress following pediatric brain injury. J Consult Clin Psychol 2006;74(3):445-454.

41. Wade SL, Taylor HG, Cassedy A, Zhang N, Kirkwood MW, Brown TM, et al. Long-Term Behavioral Outcomes after a Randomized, Clinical Trial of Counselor-Assisted Problem Solving for Adolescents with Complicated Mild-to-Severe Traumatic Brain Injury. J Neurotrauma 2015;32(13):967-975.

42. Wade SL, Walz NC, Carey J, McMullen KM, Cass J, Mark E, et al. Effect on behavior problems of teen online problem-solving for adolescent traumatic brain injury. Pediatrics 2011;128(4):e947-953.

43. Wade SL, Walz NC, Carey J, McMullen KM, Cass J, Mark E, et al. A randomized trial of teen online problem solving: efficacy in improving caregiver outcomes after brain injury. Health Psychol 2012;31(6):767-776.

44. Fortier MA, Bunzli E, Walthall J, Olshansky E, Saadat H, Santistevan R, et al. Web-based tailored intervention for preparation of parents and children for outpatient surgery (WebTIPS): formative evaluation and randomized controlled trial. Anesth Analg 2015;120(4):915-922.

45. Marsac M, Hildenbrand A, Kohser K, Winston F, Li Y, Kassam-Adams N. Preventing posttraumatic stress following pediatric injury: A randomized controlled trial of a web-based psycho-educational intervention for parents. J Pediatr Psychol 2013;120(4):915-22.

46. Newton KT, Ashley A. Pilot study of a web-based intervention for adolescents with type 1 diabetes. J Telemed Telecare 2013;19(8):443-9.

47. Chan DS, Callahan CW, Hatch-Pigott VB, Lawless A, Proffitt HL, Manning NE, et al. Internet-based home monitoring and education of children with asthma is comparable to ideal office-based care: results of a 1-year asthma in-home monitoring trial. Pediatrics 2007;119(3):569-578.
